# ApoE transports amylin from blood to brain influencing brain β‐amyloid level

**DOI:** 10.1002/alz70855_105601

**Published:** 2025-12-24

**Authors:** Noah S Leibold, Velmurugan Gopal Viswanathan, Deepak Kotiya, Laura Radulescu, Ravichandra S Davargaon, Cate Conner, Nirmal Verma, Florin Despa

**Affiliations:** ^1^ University of Kentucky, Lexington, KY, USA; ^2^ University Of Kentucky, Lexington, KY, USA

## Abstract

**Background:**

The APOE ε4 allele is the largest genetic risk factor for Alzheimer's disease (AD) and is associated with an earlier onset of AD. Amylin, a centrally acting neuroendocrine hormone co‐secreted with insulin, is elevated in blood in AD patients and forms neurotoxic amyloid plaques with β‐amyloid. Intravenous infusion of apoE4 in rats expressing human amylin increased brain amylin accumulation and revealed that amylin binds apolipoproteins *in vivo*, in our previous study. Further, we demonstrated that increased blood amylin disrupts brain β‐amyloid efflux. Here, we tested the hypothesis that apoE‐amylin molecular complexes provoke apoE4‐associated neuropathology in AD.

**Method:**

Mice double transgenic for human amylin and apoE3 or apoE4 (E3HIP, E4HIP), or apoE‐knockout (EKO‐HIP) were generated; mice with human apoE3 or E4 with mouse amylin (non‐amyloidogenic) were negative controls. Behavior tests were conducted, and brains were collected for immunoprecipitation and proteomic analyses using ELISAs. Proximity ligation assays (PLAs) were conducted to confirm colocalization of amylin‐apoE molecular complexes in brain parenchyma.

**Result:**

Novel object recognition tests suggest reduced recognition memory in E4HIP mice compared to E3HIP counterparts. Despite lacking amyloid processing mutations, E4HIP mice had significantly increased parenchymal amylin and Aβ compared to E3HIP and EKO‐HIP littermates. Brain and peripheral apoE and blood amylin levels were consistent between E3HIP and E4HIP. E3HIP and E4HIP mice had dramatically increased parenchymal amylin and Aβ, compared to mice expressing human apoE3 and apoE4 with mouse amylin. EKO‐HIP brains showed no parenchymal amylin or Aβ accumulation. Immunoprecipitation experiments from E3HIP and E4HIP brain tissue homogenates revealed apoE isoform‐dependent affinities for amylin. PLA detected robust colocalization of apoE‐amylin complexes throughout brain parenchyma in E3HIP and E4HIP mice.

**Conclusion:**

Human amylin forms parenchymal complexes with apoE, exacerbating Aβ pathology, especially in apoE4^+^ mice. In the presence of non‐amyloidogenic (mouse) amylin, apoE4 does not lead to Aβ or amylin accumulation. The lack of amylin and Aβ in EKO‐HIP brains implicates apoE as a molecular carrier for both proteins at the blood‐brain barrier. Immunoprecipitation results suggest differential strength of apoE‐amylin interactions as a result of apoE isoform. Future studies investigating the specific mechanisms regulating amylin‐apoE‐Aβ interactions are warranted.

